# Toward Genome-Based Selection in Asian Seabass: What Can We Learn From Other Food Fishes and Farm Animals?

**DOI:** 10.3389/fgene.2021.506754

**Published:** 2021-04-21

**Authors:** László Orbán, Xueyan Shen, Norman Phua, László Varga

**Affiliations:** ^1^Reproductive Genomics Group, Temasek Life Sciences Laboratory, Singapore, Singapore; ^2^Frontline Fish Genomics Research Group, Department of Applied Fish Biology, Institute of Aquaculture and Environmental Safety, Hungarian University of Agriculture and Life Sciences, Keszthely, Hungary; ^3^Tropical Futures Institute, James Cook University, Singapore, Singapore; ^4^Institute of Genetics and Biotechnology, Hungarian University of Agriculture and Life Sciences, Gödöllõ, Hungary; ^5^Institute for Farm Animal Gene Conservation, National Centre for Biodiversity and Gene Conservation, Gödöllõ, Hungary

**Keywords:** marker-assisted selection, genome-wide association study, transcriptome, genome sequencing and assembly, selected lines, aquaculture, marine predator, teleost

## Abstract

Due to the steadily increasing need for seafood and the plateauing output of fisheries, more fish need to be produced by aquaculture production. In parallel with the improvement of farming methods, elite food fish lines with superior traits for production must be generated by selection programs that utilize cutting-edge tools of genomics. The purpose of this review is to provide a historical overview and status report of a selection program performed on a catadromous predator, the Asian seabass (*Lates calcarifer*, Bloch 1790) that can change its sex during its lifetime. We describe the practices of wet lab, farm and lab in detail by focusing onto the foundations and achievements of the program. In addition to the approaches used for selection, our review also provides an inventory of genetic/genomic platforms and technologies developed to (i) provide current and future support for the selection process; and (ii) improve our understanding of the biology of the species. Approaches used for the improvement of terrestrial farm animals are used as examples and references, as those processes are far ahead of the ones used in aquaculture and thus they might help those working on fish to select the best possible options and avoid potential pitfalls.

## Introduction

The combined effects of rapidly increasing human population, growing need for seafood products, constraints on freshwater use and depleted marine fish populations (Lotze et al., 2018) exert an enormous pressure onto our aquaculture production (for review see Ahmed and Thompson, 2018). More seafood needs to be produced using less water and during a shorter period of time. This requires effective and predictable production processes that in turn necessitate the use of selected elite lines with improved performance.

With their species count exceeding 32,000; fishes form by far the largest clade among vertebrates (Nelson et al., 2016). According to rough estimates, less than one percent of them have been utilized in mid- to large-scale production and selection programs exist only for 1–2 dozen species. Most of these species are from temperate waters; their selection has been reviewed in the literature several times during the last decades [for reviews see, e.g., Yanez et al. (2015) and Abdelrahman et al. (2017)].

Asian seabass (*Lates calcarifer*, Bloch 1790; for the Latin names of fishes and other aquatic organisms mentioned in the review see Supplementary Table 1) is a large-bodied, tropical marine teleost, native to the Indo-Pacific region. Its farming has originally been developed in South-East Asia, Australia and Papua New Guinea (Cheong, 1989). Besides being one of the most popular food fish species for the above regions, Asian seabass has also entered the seafood markets of Europe and the United States at the end of the last century (Pierce, 2006). Currently, the majority of commercial production of the species is primarily done by small family-owned and mid-sized farms, using brooders collected either from the sea or unknown sources.

This review was written to describe the current status of the process that was initiated nearly 15 years ago for the development of elite Asian seabass lines using cutting-edge tools of genomics. We start by providing a general introduction of the available genomic platforms and selection strategies and their use for the improvement of land-based farm animals. Then we introduce the selection strategy used for improving the Asian seabass lines and genomic resources and platforms generated for their use for the development of elite lines and for advanced studies of the biology species. Finally, we provide a few practical advices for additional improvements in Asian seabass selection in the future and list a few considerations for aquatic selection programs in general.

## Application of Genomics in the Selection of Terrestrial Farm Animals and Fishes

Genomics studies the whole genome at various levels instead of examining individual genes. While this terminology appeared over 30 years ago, this area of research reached its full potential in the current genomic era through the utilization of numerous technological and methodological inventions. The sequencing and assembly of human (Lander et al., 2001; Venter et al., 2001) and mouse (Waterston et al., 2002) genomes were followed by those of major livestock species [see e.g., chicken (International Chicken Genome Sequencing Consortium, 2004); dog (Lindblad-Toh et al., 2005); cattle (Bovine Genome Sequencing Analysis Consortium et al., 2009); horse (Wade et al., 2009); pig (Groenen et al., 2012); goat (Dong et al., 2013); and sheep (Jiang Y. et al., 2014)]. The current status of the reference genomes is summarized in Georges et al. (2019). During this period, new high-throughput technologies, such as Next-Generation Sequencing (NGS) (for review see Goodwin et al., 2016) and Single Nucleotide Polymorphism (SNP) arrays (Chee et al., 1996; Wang et al., 1998; LaFramboise, 2009), also emerged and enabled the application of three new genomic strategies: Genome-Wide Association Studies (GWAS), Selective Sweep(/Signature) Mapping and a new genome-based selection scheme: Genomic Selection (GS). There are several commercially available SNP chips for the major livestock species with different (high, medium and low) marker densities (reviewed in Nicolazzi et al., 2015). Whole genome assemblies and SNP arrays of different fish species are overviewed in detail in three recent reviews (Abdelrahman et al., 2017; Houston et al., 2020; You et al., 2020).

### Genome-Wide Association Studies

A Genome-Wide Association Study is an advanced genetic mapping approach, which utilizes the accumulation of recombination events occurring over several generations during population history from the time the mutation arose in the common ancestor, instead of limited number of crossing overs occurring in classical linkage analysis (Hirschhorn and Daly, 2005). As revealed by high-resolution genomic techniques, recombination events are clustered to hotspots across the genome and their occurrence is very rare in the intervening haplotype blocks. Thus, the association of alleles of the physically linked loci in these blocks is non-random, which is the classical definition of Linkage Disequilibrium (LD) (Kruglyak, 1999).

The prerequisite of GWAS is a dense set of markers covering the whole genome with uniform spacing as the presence of markers on each haplotype-block assures the detection of LD with the causative variants. The number of SNPs required depends on the LD pattern of the population, which is shaped by different demographic and genetic forces such as effective population size, admixture, selection, mutation, recombination etc. (reviewed in Qanbari, 2019). While the LD is relatively short range in humans and needs high-density SNP chips for GWAS, farm animal populations have generally longer range genome-wide LD, requiring lower marker density (Andersson, 2009). The sufficient density of markers can be reached by SNP chips (Matukumalli et al., 2009), or by Genotyping-by-Sequencing (GBS) methods (Elshire et al., 2011; Gorjanc et al., 2015), or by low-coverage genome sequencing (Pasaniuc et al., 2012). Thus, GWAS can assure high-resolution mapping, provided that the experimental arrangements take into consideration several factors affecting the success of the association studies, such as size and genetic structure of the population (admixture should be avoided), the marker density of the SNP chip, statistical model as well as technical artifacts (Hirschhorn and Daly, 2005; Altshuler et al., 2008; Goddard and Hayes, 2009; Hill, 2012; Wang G. D. et al., 2014).

Numerous GWAS designs were applied in livestock species (Goddard and Hayes, 2009; Fan et al., 2010). A good example to illustrate the power of this approach is the mapping of the white-spotting (S) locus in the dog. Using a two-stage, long LD, within-breed GWAS and an ancestral, short LD, across-breed fine-mapping approach, it was possible to identify the gene (MITF; microphthalmia-associated transcription factor) harboring the causative mutation, despite of the fact that the mapping population contained 127 dogs from two breeds only (Karlsson et al., 2007). Association mapping is effective not only in the case of monogenic traits [for example myostatin affecting racing performance in horses (Hill et al., 2010)], but it is able to reduce substantially QTL (Quantitative Trait Loci) regions of different complex traits of economic importance. Some recent GWAS examples in terrestrial farm animals are: bovine – fertility traits (Ma et al., 2019); sheep – morphological and agronomic traits (Li et al., 2020); pig – candidate genes for backfat and intramuscular fatty acid composition (Crespo-Piazuelo et al., 2020); horse – athletic performance related genes (Littiere et al., 2020).

In fishes, the major fields of interest in GWAS are growth in Atlantic salmon (Gutierrez et al., 2015), rainbow trout (Reis Neto et al., 2019), common carp (Su et al., 2020); muscle and filet yield in rainbow trout (Gonzalez-Pena et al., 2016; Salem et al., 2018; Ali et al., 2019) and Atlantic salmon (Yoshida et al., 2017); as well as disease resistance in channel catfish (Geng et al., 2015) and Atlantic salmon (Robledo et al., 2018; Hillestad et al., 2020).

### Selection Sweep Mapping

High-density genome-wide marker sets allow for the identification of signatures of selection (Sabeti et al., 2007). These specific footprints are formed under a continuous selection pressure induced by the breeders unintentionally by favoring a particular advantageous phenotypic trait. According to the selective sweep theory, when a beneficial mutation arises in a population and a strong selection pressure increases its frequency, it is rapidly fixed in the population. However, neighboring hitchhiker alleles in LD with the mutation will undergo also the same process, since recombination will not have enough time to separate them. The resulting specific Runs-of-Homozygosity (ROH) is a characteristic sign of selection together with the higher frequency of otherwise rare alleles and the marked differences between marker allele frequencies in the region compared with a population without selection (Nielsen et al., 2007; Andersson, 2012; Vitti et al., 2013; Qanbari and Simianer, 2014). In addition to natural selection on the evolutionary scale (Nielsen, 2005), signals of recent artificial selection for economic traits under positive selection can leave footprints in the genome even over a few generations (Sabeti et al., 2007; Zhao et al., 2015). Similarly to GWAS, selection mapping is also an effective strategy toward the identification of beneficial mutations the population/breed was selected for. These two mapping strategies can be combined to validate the candidates determined with one or the other (Akey et al., 2010; Schwarzenbacher et al., 2012; Qanbari and Simianer, 2014; Zhao et al., 2015; Johnsson, 2018).

While GWAS can be characterized as a forward genetic approach (phenotype-to-genotype), selection mapping can be considered as a reverse genetic procedure (genotype-to-phenotype). The latter analyses the genomic data representing the footprints of selection only, whereas the phenotype is the overall phenotype of the population/breed (Ramey et al., 2013; Bomba et al., 2015). Contrary to GWAS, the advantage of selection mapping is that mutations already fixed in the population can also be mapped by this approach (Qanbari and Simianer, 2014). On the other hand, its disadvantage is that demographic factors (bottlenecks and founder effect) of the population might generate false positive results (Cutter and Payseur, 2013; Qanbari and Simianer, 2014; Zhao et al., 2015).

There are numerous selection mapping publications on livestock species [e.g., dog (Akey et al., 2010); pig (Rubin et al., 2012); dairy and beef (Zhao et al., 2015); horse (Gurgul et al., 2019); for review see Qanbari and Simianer, 2014].

The applicability of selection mapping in fishes was reviewed by Lopez et al. (2014). Whole genome sequencing or SNP genotyping were performed on several different farmed species. The traits to be mapped with this approach were usually of great economic importance in fish farming. Candidate genes within the ROH regions identified were related to (i) rapid growth, non-grilsing, development, behavior, olfaction, immune system, and reproductive traits in Atlantic salmon populations (Gutierrez et al., 2016; Veale and Russello, 2017; Lopez et al., 2019; Naval-Sanchez et al., 2020); (ii) growth, early development and immunity traits in Nile tilapia (Cadiz et al., 2020); and (iii) thermal adaptation in redband trout (Chen et al., 2018).

How can results of GWAS and selection mapping be utilized by the breeders? First, when the causative mutation is mapped to a narrow chromosomal region, the nearest markers can be used to test its transmission to the progeny (see below), while after its identification, straightforward direct gene tests can already be performed. The “side-product” of these high-throughput mapping procedures is the whole-genome SNP polymorphism data, which can be used to estimate the different population genetic parameters. At the same time, the genome-wide level of homozygosity can be also determined from a single animal and the individual inbreeding (coefficient) can be calculated. This value can be used in mating plans to avoid reductions of fitness traits in the subsequent generations (Keller and Waller, 2005; Kristensen et al., 2010; Kardos et al., 2016).

### Marker-Assisted Selection

Marker-Assisted Selection (MAS) was the only method available for genome-enabled selection in the pre-genomic era (Neimann-Sorenson and Robertson, 1961; Smith, 1967). Unfortunately, its impact has not been as high as expected, since conventional microsatellite-based linkage mapping could not uncover such tight marker-trait associations as those detected later by SNP chips. MAS characteristically focuses on a single or a few significant marker–trait association(s). It requires a preceding mapping analysis to find the closest markers, preferably on both sides of the unknown mutation. Transmission of the target allele can be thus followed by genotyping of the marker alleles in the subsequent generations. The bracket configuration ensures double-direction control the recombination events, which can destroy LD among the marker and trait alleles (Soller and Andersson, 1998; Spelman and Bovenhuis, 1998; Dekkers, 2004; Goddard and Hayes, 2009). The first significant MAS reports on livestock species were published in the early 90’s [e.g., pig halothane locus RYR1-(Fujii et al., 1991); booroola FecB mutation (Montgomery et al., 1993), polled locus (Georges et al., 1993), among others].

One of the most successful examples of QTL analysis applied to selective breeding is the case of increased tolerance against infectious pancreatic necrosis (IPN) in Atlantic salmon. IPN is a viral disease that causes high losses in the stocks of salmon and rainbow trout farms worldwide. As a result of extensive research efforts, it was found by two research teams that a major QTL explains 50.9% of the phenotypic variance for resistance against IPN (Houston et al., 2008; Moen et al., 2009). An LD-based test was used to deduce the QTL allele, and the test was then utilized to produce salmon lines with increased tolerance against IPN, causing a dramatic decrease in the frequency of IPN infections in the industry. The QTL was mapped by whole-genome sequencing of individuals carrying deduced QTL and it contained an epithelial cadherin (*cdh1*) gene, that is likely to contribute to the entrance of virus into the cells (Moen et al., 2015).

### Genomic Selection

The invention of GS resulted in a paradigm shift by raising the genome-enabled selection to genomic level (Meuwissen et al., 2001; Goddard and Hayes, 2009). Its concept assumes, that having a marker set covering the whole genome densely and evenly, all QTLs are supposed to be in LD with at least one marker. As such, this procedure does not require a mapping step. GS is a two-stage procedure. First, a reference population (RP) has to be assembled from animals with highly accurate phenotypic values preferably for several different traits of importance and assayed with the SNP chip connecting phenotypic information to the genotypic values (Hayes et al., 2013). The summed effect of each SNP genotype present in the RP can be estimated to set up a prediction equation for the particular trait. This equation can then be used in the second, validation step to determine the genomic estimated breeding value (GEBV) of selection candidates that have SNP genotype, but no phenotypic records (Stock and Reents, 2013). Based on GEBV, candidates are ranked and the animals with the highest scores will be selected for breeding the subsequent generations (Dekkers, 2012; Pryce and Daetwyler, 2012; Schefers and Weigel, 2012; Hayes et al., 2013).

All domestic species have unique species-specific characters (e.g., generation interval, individual value, breeding program, population management, accuracy of phenotypic measures, genomic resources, costs of the tests and returns on investments, etc.) making them more or less suitable for GS. Since SNP chip analysis is an expensive procedure, the individual price of selected animals and cost effectiveness of the whole procedure will determine the applicability of GS (Goddard and Hayes, 2009; Hayes and Goddard, 2010).

Dairy cattle occupies a special position among livestock species since adoption of GS was pioneered by the dairy industry, and this is the most successful application of the methodology so far due to the specialties of large-scale milk production (Wiggans et al., 2017). First, the breeding value of a bull can be determined on the basis of milk production of his daughters by traditional progeny testing, which is a long and very expensive procedure, since the generation interval is at least 5 years (Eggen, 2012). At the same time, the individual value of the animals is relatively high. Since the gradually decreasing SNP-genotyping costs dipped below the overall costs of progeny testing, a paradigm shift occurred in the practical breeding from the conventional selection toward GS (Schefers and Weigel, 2012). The main determinant of accuracy is the size of RPs, which were thousands of animals at the beginning and subsequently collaborations were organized to enlarge the size of the international RPs as much as possible (Lund et al., 2011). GS turned out to be so successful in cattle breeding, that progeny testing is continuously losing its importance in the breeding process (Boichard et al., 2015). GS has been proven to be more advantageous than classical selection based on phenotype and pedigree information, especially for those economic traits that are difficult or expensive to measure, like fertility, disease resistance, etc. (Hayes et al., 2013). Moreover, GS-based selection can be performed at early age. All three main constituents can modulate effectively the rate of genetic gain (ΔG = irσ/L, where “Δ*G*” is the genetic gain per year, “*i*” is the intensity of selection, “*r*” is the accuracy of the selection, “σ” is the genetic standard deviation and “*L*” is the generation interval) in dairy GS. Intensity and accuracy can be increased substantially, while generation interval can be reduced drastically, facilitating widespread application of the method all over the world (Hayes et al., 2013). Several factors, including incomplete pedigree data, low/moderate quality and quantity of phenotypic data, lower accuracy of genomic predictions and infrastructure, hamper the adaptation of GS for beef cattle. Many production traits can be measured directly on selection candidates without the need of progeny testing, thus the economic advantage is lower than in the dairy cattle (Montaldo et al., 2012; Van Eenennaam et al., 2014; Meuwissen et al., 2016).

In prolific livestock species (e.g., pig and chicken) the opportunities offered by GS are not as straightforward as in the case of dairy cattle (Blasco and Toro, 2014). In the breeding pyramid structure of pig and chicken, GS seem to be profitable only at the top level (Goddard and Hayes, 2009; Stock and Reents, 2013; Van Eenennaam et al., 2014; Jonas and de Koning, 2015; Knol et al., 2016). Since the generation interval is *ab ovo* short for these species, the potential of GS can be exploited to improve the accuracy of selection (Stock and Reents, 2013). In spite of the continuously decreasing SNP genotyping price, a highly cost-effective strategy is needed to assure the widespread implementation of GS in these species (Wolc et al., 2016).

Fish breeding faces the same problems in this regard since the breeding scheme, the typical farm size and the average individual value are similar to those of pig and chicken. Due to these reasons, only the most affordable approaches (e.g., low density genotyping) can be applied routinely even in high-value fish species (Lillehammer et al., 2013; Kriaridou et al., 2020). Reduction of the density and thus the price of the SNP chip seems to be a key factor for the widespread applicability of GS in aquaculture species. Contrary to the terrestrial livestock species, it was astonishing that reduction of marker density to 1000–2000 SNPs yielded similar selection accuracies to those of the high-density arrays (see section “A few considerations for food fish selection programs” for further details). High fecundity, large family sizes of fishes and the GS practice applied explain this observation. The tremendous number of siblings produced from a single spawning can be divided to RP and selection candidates. Huge genomic segments shared between these two closely related populations ensure the reliable genome-wide LD representation by even a low density array (Kriaridou et al., 2020). GS has been applied in Atlantic salmon for growth traits (Tsai et al., 2015), for sea lice resistance (Houston et al., 2014; Tsai et al., 2016); in rainbow trout for bacterial cold water disease resistance (Vallejo et al., 2016, 2017), but examples can be also found among the high-volume species like common carp (Palaiokostas et al., 2018), or channel catfish (Garcia et al., 2018); for reviews see Houston et al. (2020) and You et al. (2020). For the rest of the fish species GS remains a future possibility for the time being.

## A Selection Program for a Tropical Predator, the Asian Seabass

### Traditional Aquaculture of Asian Seabass in South-East Asia and Australia

At the turn of the millennium, the majority of Asian seabass farms operating in South-East Asia consisted of small, coastal units (Cheong, 1989). Some of these farms had their own broodstock (typically a mixture of wild-caught brooders with those obtained from other farms), whereas others relied entirely on larvae purchased from commercial facilities. In both cases, no information was available on the genetic relatedness of brooders or the offspring individuals.

In South-East Asia, most seabass farms perform their whole operation (i.e., spawning, larval rearing and grow-out) in full seawater. Breeding groups are usually acclimated at one female to two males ratio while preparing for spawning in sea cages (Chou and Lee, 1997) or cement/fiberglass/canvas tanks at temperatures ranging from 28–30°C. Brooders are typically kept at low density and fed with a high protein-based commercial diet, often fortified with vitamins. Groups are acclimated until the hierarchy among the brooders is formed and there are no more fights among them. Even for brooders kept in building-based tanks with no direct connection to the sea, spawning usually happens close to the new moon or full moon period. Most facilities increase the efficiency of spawning by administering either luteinizing hormone-releasing hormone analog (LHRHa) or human choriogonadotropin (hCG) injections to the brooders (Tucker et al., 2002). Injections are usually timed one night before the expected spawning event.

A female brooder spawns up to a million of eggs over a period lasting up to 2 days. Fertilized eggs either float on the surface or suspend in mid-water, whereas unfertilized ones sink to the bottom. Fertilized eggs are scooped or siphoned into an egg collection chamber with a stocking density of less than 2,000 eggs per liter. Within 24 h of spawning, most fertilized eggs hatch into larvae (Rimmer and Russell, 1998).

At 1 day post-hatching (dph), newly hatched larvae are transported to culture tanks where a mixture of various phytoplankton species (typically Nannochloropsis and Isochrysis, in some cases supplemented with vitamins), are added to a to the water. Rotifers are introduced to the culture tanks to induce the first feeding of larvae around two dph and remain the main food source until 15 dph. Freshly hatched Artemia (with or without enhancement) and dry feed are introduced at 12 and 15 dph, respectively (Rimmer et al., 1994). Typically, dry feed is mixed with artemia and fed to the fry through several days in increasing proportion.

Cannibalism among seabass fries increases substantially during this period, as individuals that weaned over to dry feed grow substantially faster than those that still prefer artemia. Losses to cannibalism can be reduced, but not eliminated by feeding to satiety. Once the fries are fully weaned, fish graders are used to separate the fry/juveniles into different size groups that must be housed in different tanks (alternatively, the groups containing largest and smallest individuals can be terminated, if their size is small). Gradings are performed at every third-fourth day between 20 and 60 dph.

Most farms introduce fingerlings into the sea cages around 30 dph as only a few can afford to keep them under sheltered conditions until they can be vaccinated against known bacterial diseases. The conditions among which the fingerlings are kept in sea cages allows for constant access of various harmful pathogenic agents to them. As a result, typical survival rates of unvaccinated fingerlings to marker size (600–900 g/individual) are highly variable, often falling below 5–10% of the individuals introduced into the sea cage or grow-out tank. Farms with full-scale nursery operations tend to keep their fingerlings at their land-based facilities until they can be vaccinated (3–4-inch size) prior to their introduction to the sea cages.

In Australia and some farms of Malaysia and Thailand, Asian seabass is grown in freshwater, water obtained from drilled wells or brackish water (Alain Michel, personal communication). Their fish are often grown to 3–5 kg size and sold as chilled filets.

More detailed information on traditional culture conditions can be found in the reviews of Copland and Grey (1986) and Russell and Rimmer (2004) whereas modern practices are described in full details in the book edited by (Jerry, 2014).

With the advancement of aquaculture technologies in the world, food fish industry in Asia was poised for a period of substantial growth at the turn of the millennium. However, a greater demand for more harvested fishes in turn increased the need for quality fingerlings. In 2004, in collaboration with internal (the Yue team, Temasek Life Sciences Laboratory) and external partners (e.g., Agri-Food and Veterinary Authority of Singapore), we have started a selection program to develop elite lines of Asian seabass that produce faster-growing and more robust fingerlings for the aquaculture industry. In parallel, we also aimed to generate genomic tools and platforms to support the selection program. The following sections describe the progress made, the genetic and genomic platforms generated and some of the problems as well as opportunities that have surfaced over the past one and a half decades.

### The Protocol Applied for Asian Seabass Selection

**FIGURE 1:**
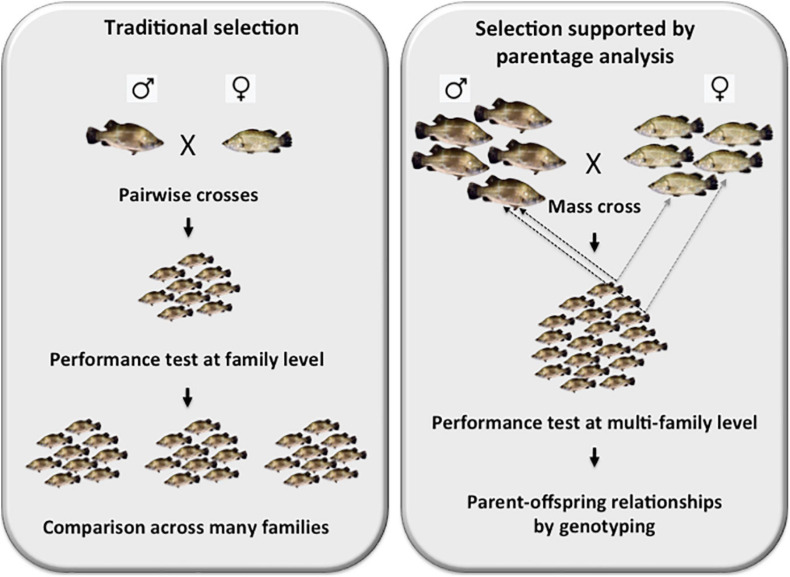
Schematic representation of the differences between a family-based traditional selection *vs.* a modern selection program supported by parentage analysis. The left panel depicts a family-based selection, where selected pairs of brooders are crossed, their offsprings are raised together and their performance analysis is performed by comparing siblings within the family. In this approach, many families must be analyzed individually and often repeatedly as environmental effects can have a substantial effects on the results of these comparisons. On the right panel, the essence of a modern selection program supported by parentage analysis based on genotyping with polymorphic DNA markers is shown. In this protocol, mass crosses involving up to 25 males × 25 females are performed. Following the validation of multi-family contribution by microsatellite-based genotyping, offspring are raised together to the age, when their performance can be assessed. The best-performing offspring individuals are then fin-clipped and genotyped with multiplexed microsatellite sets to reveal their parents. The brooders are then crossed again to (i) validate the results of the first cross; and (ii) possibly generate new families with even better results.

In order to set up the founding broodstock population, over 700 wild-caught young Asian seabass adults were sourced from three different locations: Indonesia, Malaysia and Thailand. They were analyzed with polymorphic microsatellites isolated earlier in our laboratory (Yue et al., 2001; Yue et al., 2002) that according to our knowledge were the first such set of markers for the species. Later, others have also contributed to the collection (Sim and Othman, 2005; Abdul Rahman et al., 2012). As the results of a phylogeographic analysis based on microsatellites have shown lack of clear separation among the above three major groups (Yue et al., 2009), the geographic origin of broodstock individuals was not taken into consideration during the selection process. Subsequently, additional, highly polymorphic microsatellite markers were isolated from the genome of Asian seabass by the Yue team (Zhu et al., 2006, 2010). Following their characterization, a selected set was combined into multiplexes based on their average allelic size and the width of their allelic range. For the analysis of our brooders and offspring a 9-plex microsatellite set that allowed for an efficient parent-sibling matching of >95% in most crosses, was used (Liu et al., 2012; Yue et al., 2012). In those rare cases, when the efficiency has dropped substantially below this value, several other 5- and 4-plexes could be utilized for obtaining additional genotypes. The development of these multiplex genotyping sets was a crucial step, as it allowed the upgrade from a slow and laborious family-based selection strategy to a more effective process based on testing the origin of best performing offspring from mass crosses using molecular parent-offspring matching as described above (Figure 1). In addition to their essential role during the initial setting up of broodstocks (Yue et al., 2009), these microsatellite sets can also be used for surveying the status of genetic diversity and the level of possible inbreeding in existing broodstocks (Loughnan et al., 2016).

The selection process was based on mass crosses containing 8–50 brooders. We have used two different sizes of spawning groups depending on the needs of the project and availability of resources. In the small groups, we typically placed 8–12 brooders into a 7 m^3^ cylindrical indoor tank, whereas large groups containing 15–40 brooders spawned in 60 m^3^ indoor concrete cylinders. Spawning groups were formed to keep the number of combinations high and minimizing the allelic losses across generations in order to slow down the decrease of genetic variability within the breeding nucleus as much as possible. The selected group of brooders was allowed to settle for a couple of weeks, and then they were prepared for spawning. The process of spawning typically started during the night of full or new moon and lasted for several days. Representative samples of larvae at 1–3 days post-hatching (dph) were collected and used for estimating of the contribution of families to the mass offspring. This allowed for the identification of those offspring batches (i.e., those produced by a particular set of brooders in one spawning event), where the minimally required number of contributing families (at least three families in small groups and at least five in large groups with >3% of larvae per family) were not reached. Upon confirmation, these batches were terminated and those crosses were repeated at a later stage.

Batches with the required minimal number of contributing families were raised indoor in cylindrical tanks of increasing volume. Larvae, fingerlings and juveniles were grown at optimized conditions, since the standard larviculture protocol used in the aquaculture industry had to be modified for the faster-growing selected larvae. Larval offspring from selected breeding pairs usually reached the size range suitable for Artemia (brine shrimp) consumption substantially earlier than the age typically used by the industry. The quantity of live feed also had to be raised in order to satisfy the increased appetite of larvae. Typically, seabass fingerlings are fed 2–3 percentage of their body weight daily in 3–4 feedings, but for the offspring of selected brooders, a “feed till they are full” method was introduced. At such conditions, seabass fingerlings consumed up to 8% of their body weight per day.

The relative contribution of environmental and genetic factors to the appearance of cannibalistic behavior in fishes is not well understood (for review see Hecht and Pienaar, 1993). In aquaculture practice, cannibalism is usually reduced by proper feeding and regular size selection (grading) performed by carefully shifting the fingerlings through mechanical sieves, a rather labor-intensive process. We expected a that the gap between two gradings could be extended for the selected fish as a result of their more uniform size. However, to our surprise, our faster-growing, selected fingerlings required more frequent grading – every 2-to-3 days – resulting in increased need for manpower and effort.

During grading, the largest individuals with size several fold higher than the average of the batch (typically <3% of the population) were usually separated from the rest of the fingerlings. Since this group is expected to contain the most cannibalistic individuals, thus, similarly to most commercial farms, we have typically sacrificed these individuals. Although, we might have lost a portion of the combined growth potential of the batch that way, an additional reduction in the chance of cannibalism appeared to justify this practice.

At 90 dph, the survivors were subjected to detailed phenotypic analysis, including the determination of morphometric parametres. Parent-sibling connections were determined by genotyping and selection was performed by limiting the number of individuals kept for the next generation from the same family to minimize allelic loss and maintaining a widely based contribution of the families to the next generation.

According to our knowledge, several other selection programs have been or are being performed on the Asian seabass in the Asia-Pacific region: at least three in Australia (Robinson et al., 2010; Macbeth and Palmer, 2011; Domingos et al., 2014; Khang et al., 2018), one in Indonesia (Michel, 2017) and one in Malaysia.

### Development of New Genetic and Genomic Platforms for the Asian Seabass

In addition to the microsatellites, we and our collaborators have developed several genetic and genomic platforms in order to help current and future improvements of the selection process (Table 1). As these platforms have become available during the selection program, they allowed us to learn more about the biology of seabass and opened up avenues toward the utilization of more modern approaches. Two genetic linkage maps have been generated by our collaborators. The first one was a low-density map with 240 microsatellite markers (Wang et al., 2007), later they increased the number of mapped markers to 790 microsatellites and SNPs (Wang et al., 2011). An effort was made to generate maps with even marker distribution, as much as possible. Subsequently, a high-density map was produced by GBS of 144 individuals from an F_2_ family that allowed the placement of 3,321 SNPs onto the map (Wang et al., 2015). Three BAC libraries were also produced: the first, in-house library contained over 49,000 clones with an average insert size of 98 kb (Xia et al., 2010), whereas the second and third commercial ones generated by Amplicon Express (Pullman, WA, United States) had ca. 115 kb average insert size and over 73,000 clones combined. The first library was used for targeted analysis of a QTL located on linkage group two and associated earlier with increased growth (Wang et al., 2011). Several SNP markers within three candidate genes were identified that could potentially be used as markers for selecting fast-growing seabass fingerlings (Shen et al., 2016). The other two BAC libraries were utilized during the quality control of the assembled genome (see below).

**TABLE 1:** Summary of genomic tools developed for Asian seabass.

Genomic tools	Year published	Applications	References
Polymorphic microsatellites	2001	Initial genetic analysis of natural populations and farmed stocks	Yue et al., 2001, 2002
Genetic linkage mapping	2007	Mapping QTLs for increased growth rate	Wang et al., 2007, 2011, 2015
BAC libraries	2010	Validation of genome assembly; Targeted analysis of a QTL associated with increased growth	Xia et al., 2010; Shen et al., 2016
Expressed sequence tags	2010	Early analysis of gene expression	Xia and Yue, 2010; Xia et al., 2011
Multiplexed microsatellites	2012	Parent-sibling matching	Liu et al., 2012; Yue et al., 2012
Gene set for qPCR analysis	2014	Identification of DEGs during natural sex reversal	Ravi et al., 2014
Expression microarray	2014	Monitoring transcriptomic responses to vaccination and/or infection; Expression analysis of gonads during natural sex reversal	Jiang J. et al., 2014; Jiang, 2014
*De novo* transcriptomes	2015	Support for reference genome assembly and gene annotation	Thevasagayam et al., 2015
Repeat inventory	2015	Identification of new markers	Kuznetsova et al., 2014
Draft genome	2015	Initial information about the genome	Domingos et al., 2015
High-quality reference genome	2016	Detailed, chromosome-level information about the genome	Vij et al., 2016
Re-sequencing of genome variants	2016	Validating the existence of a species complex	Vij et al., 2016
Genotyping-by-sequencing	2017	Mapping of QTL associated with increased resistance against iridovirus	Wang et al., 2017
B chromosome	2018	Additional analysis of the species complex	Komissarov et al., 2018
Metabiome	2020	Skin microbiome of healthy and infected seabass	Miyake et al., 2020

*BAC, bacterial artificial chromosome; DEG, differentially expressed gene; qPCR, quantitative PCR; QTL, quantitative trait locus.*

In 2011, we have obtained funding for creating an assembled and annotated genome for Asian seabass. At that point, only collections of expressed sequence tags (ESTs) and partial transcriptomes were available for this species, later some of them were published (see e.g., Xia and Yue, 2010; Xia et al., 2011; Newton et al., 2013). We have started our work with the generation of a comprehensive *de novo* transcriptome for protein-coding genes. Three different NGS platforms (454-FLX Titanium, SOLiD 3+, and paired-end Illumina HiSeq 2000) were used to sequence RNA isolated from several organs of multiple individuals. The transcriptome assembly contained nearly 27,000 contigs with an average sequence length of 979 bp. When compared to the available Nile tilapia RefSeq dataset, over 80% of the Nile tilapia reference proteins were represented by at least one contig in the Asian seabass transcriptome (Thevasagayam et al., 2015). An expression microarray was designed on the basis of the assembled transcriptome. It contained a total of 60,080 probes, out of which approximately 28,000 were designed to match known transcripts that showed homology to the NCBI RefSeq Database through BlastX or Blastn, whereas the rest were based on unknown transcripts with no such hits. This expression array has later been used in the study of transcriptomic responses to vaccination and/or infection (Jiang J. et al., 2014) as well as changes of the transcriptomic landscape in the transforming gonad during natural sex reversal (Jiang, 2014).

Sequencing, assembly and annotation of the 670 Mb Asian seabass genome started in 2012. The specimen selected for sequencing was a partially inbred individual generated by crossing two very closely related parents originating from the South-East Asian region. Originally, we were aiming to perform a “chimeric assembly” based on the combination of a 25–20× coverage of long reads from Pacific Biosystems (PacBio), and an 80–100 coverage of short Illumina paired end reads as well as partial or full sequences from a selected set of BAC clones. Subsequently, we have modified our strategy and the eventual chromosomal level assembly was based solely on PacBio sequences, followed by genetic and optical mapping, as well as syntenic information obtained by multi-genome comparisons (Vij et al., 2016). Prior to the publication of this high-quality reference genome, a draft genome based solely on Illumina short-read sequences was also published by our Australian collaborators (Domingos et al., 2015).

The reference genome allowed us to produce a repeat inventory (Kuznetsova et al., 2014). Repeats have occupied an estimated 8–14% of the Asian seabass genome. Most were clustered in the pericentromeric regions, whereas the rest could be found in the pretelomeric regions. The Asian seabass karyotype contains 24 pairs of A chromosomes and a variable number of B chromosomes. Two of the latter were isolated by laser microdissection and sequenced. Their detailed analysis showed the presence of 75 B-chromosome-associated genes and a much lower repeat content that that of the A chromosomes [2 vs. 16.8%; (Komissarov et al., 2018)].

### Genomic Platforms Help to Improve Our Understanding of Asian Seabass Biology

Although they were developed with the primary aim of improving the selection process, the genomic platforms described above also offer excellent opportunities for detailed analysis of various aspects of the biology of Asian seabass. A few representative examples for such studies performed by us and others are provided below.

**FIGURE 2:**
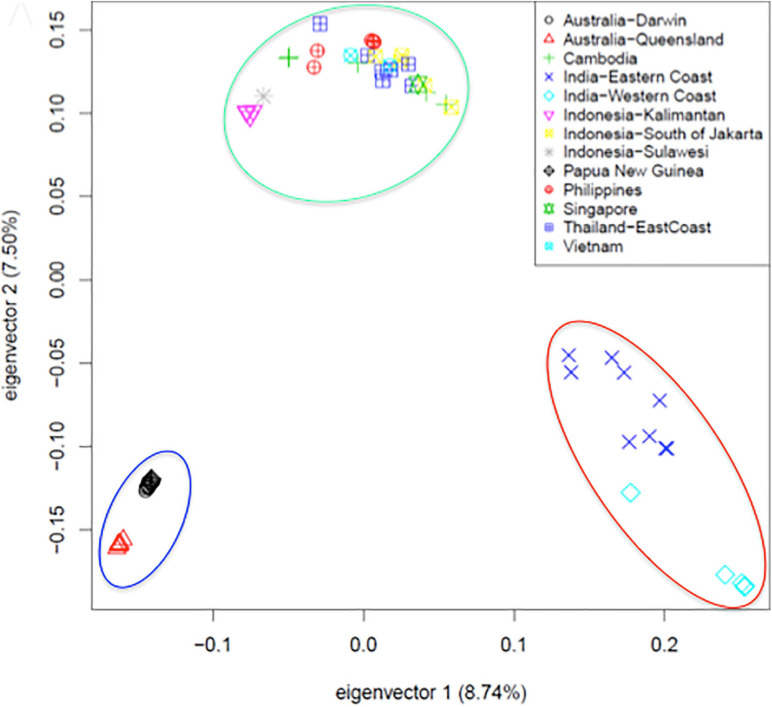
Principal component analysis of SNPs collected from 61 re-sequenced genomes supports the existence of a Asian seabass species complex and its separation into two species and a third variety [adapted from Figure 5A of Vij et al. (2016) with the authors’ permission]. Fin clip samples were collected from twelve geographical locations representing the native range of the species extending from North-Western India, through South-East Asia to North-Eastern Australia and sequenced to an average 6.7-fold coverage by Illumina short read technology. Three groups (Indian region – red ellipse, SE Asia/Philippines – green, and Australia/Papua New Guinea – blue) bearing clear allopatric signatures of separation could be observed through Principal Component Analysis. With the exception of Philippines, Vietnam and Singapore, all individuals from the remaining locations were wild-caught.

The availability of a high-quality reference genome made it possible to perform a comparative analysis of specimen originating from across the vast geographic territory occupied by Asian seabass. This was motivated by earlier reports based on morphometric analyses (Pethiyagoda and Gill, 2012; Vij et al., 2014) and phylogenetic studies based on a limited number of mitochondrial and nuclear markers (Ward et al., 2005), indicating the potential existence of more than one species of Asian seabass. Data from low-coverage whole genome resequencing of 61 individuals – together with detailed morphometric analysis - provided final proof that Asian seabass is in fact a species complex containing two different species (Indian and South-East Asian) and an Australian subspecies (Figure 2) that is in the process of splitting off from the latter (Vij et al., 2016).

The assembled transcriptome allowed for the generation of an expression microarray that was utilized for multiple purposes. One of its first uses was the detection of effects of bacterial infection onto vaccinated and un-vaccinated control specimens. Vaccinated Asian seabass juveniles were exposed to *Streptococcus iniae* at the age of 3 months and their gene expression profile in the spleen and head kidney were compared to those of sham-injected, uninfected controls. Results of comparative transcriptomic analysis showed that the response to vaccination was different between the spleens and head kidneys: an early, but transient transcriptomic change was seen in the former, whereas a delayed response in the latter (Jiang J. et al., 2014). Upon pathogen challenge, the transcriptome of spleen, but not that of head kidneys, showed a rapid response, indicating different roles for the two immune-organs in establishing a vaccine-induced disease resistance.

The same microarray was used for nutrigenomic analysis on the effects of commercial pelleted feeds onto the metabolism of Asian seabass juveniles that were selected for increased growth rate over two generations. Comparative study of the liver-based transcriptomes has identified a set of differentially expressed genes (DEGs) between pellet-fed and control (frozen baitfish-fed) individuals (Ngoh et al., 2015). Pathways of “Biosynthesis of unsaturated fatty acids” and “Steroid biosynthesis” were both enriched in pellet-fed groups in comparison to controls. There were also substantial differences among the three pellet-fed groups as well.

**FIGURE 3:**
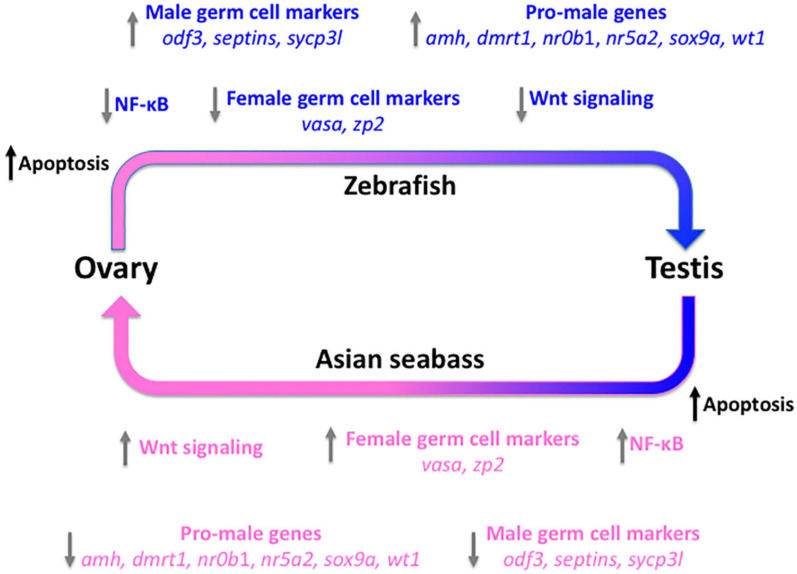
Molecular events during gonad transformation in teleosts using the zebrafish and the protandrous Asian seabass as models. All immature zebrafish develop a “juvenile ovary” before the future males undergo gonadal transformation to form the testis. Several pathways and genes have been shown to be involved in this process. We propose that many of these are also involved in the testis-to-ovary transformation of Asian seabass, despite the direction being the opposite. The arrows show the observed (zebrafish; top – blue) and/or predicted and later partially confirmed (Asian seabass; bottom – pink) differentially regulated genes or pathways during the gonadal transformation process. Apopotosis (black) is the first pathway activated at the beginning of the transformation in both systems to eliminate cells that cannot be trans-differentiated, independently from the direction. The trigger is unknown in both cases [improved Figure 6 of Ravi et al. (2014) with the authors’ permission].

We have also analyzed the transcriptome of seabass gonads during natural sex reversal. Asian seabasses are protandrous sequential hermaphrodites, they mature and breed as males, then years later they change their sex to transform into fully functional females (Moore, 1979; Davis, 1982; Guiguen et al., 1994). Thirty gonad samples were examined histologically, grouped according to their sex and staged according to gonadal maturation status. In the first round, we have analyzed a selected set of genes, including those with sex-related function and germ-cell markers. Of the 37 genes tested, over 85% turned out to be DEGs allowing us to classify the gonads analyzed according to their sex and gonadal maturation stages based on their small-scale transcriptomic profiles. Comparison to data obtained from zebrafish juveniles – which show juvenile female-to-male type gonadal transformation into future males – indicated that very similar, largely overlapping set of genes and cascades are involved in natural sex reversal independently from the direction (Figure 3; Ravi et al., 2014). According to recent data, treatment with ethinyl-estradiol through the feed can be used to promote feminization of mature Asian seabass males (Banh et al., 2020). Subsequent analysis of the process with expression microarrays (Jiang, 2014) confirmed the indication from the small-scale study. These data provide new insights into the molecular mechanisms involved in gonadal transformation of Asian seabass and likely other teleosts as well.

Intensive culture systems with large number of animals produced at limited areas offer easy opportunities of pathogen infections. This is especially true for aquatic systems, where it is very difficult to stop or even reduce the spread of diseases. Therefore, it is not surprising that viruses, bacteria and parasites pose the biggest threat to the fishes produced at commercial farms. During the second half of the last century, the only practical tools against infective diseases were vaccines and most the protective efforts were focused into their development and improvement (for review see Lafferty et al., 2015). With the introduction of genome-based approaches, the possibilities of developing selected, elite lines with increased tolerance toward certain diseases have shown substantial improvements (for review see Fraslin et al., 2020). To date, studies on identifying genetic loci associated with increased resistance to a bacterial or a viral pathogen have been conducted in several fish species of great economic value, including of Atlantic salmon (Moen et al., 2007; Gonen et al., 2015) rainbow trout (Johnson et al., 2008; Vallejo et al., 2014), Japanese flounder (Fuji et al., 2006; Wang L. et al., 2014), Atlantic cod (Odegard et al., 2010), and gilthead sea bream (Massault et al., 2011). In order to identify Asian seabass individuals with increased disease tolerance, we performed challenges on fingerlings under typical farm conditions (a complex pathogen-infected marine environment), which are expected to be directly relevant to production under commercial conditions. First, offspring groups were produced by mass-crossing selected F1 and F2 brooders. Fingerlings were grown to 2.5–3 cm size under sheltered indoor conditions, then they were transferred into raw seawater at three coastal fish farms at 30–45 dph of age. Altogether, nearly a quarter of a million fingerlings were transferred in a total of 16 batches. In most batches, the fish were naturally infected by multiple pathogens, including iridovirus, big belly disease and Vibrio sp., during the challenge period. Genotyping a subset of “sensitive” and “tolerant” individuals will allow for the assignment to their parents. Family contribution of “tolerant” individuals will be compared to those of the “sensitive” ones as well as to the initial composition of the batch at the beginning of the “field environment challenge” to identify those brooders, whose offspring tolerated the exposure to raw seawater better than the rest.

In parallel, our collaborators have performed lab-based single pathogen challenges on Asian seabass fish individuals from a selected set of families. Several QTLs (Proportion of Variance Explained, or PVE from 7.8 to 11.0%) associated with viral nervous necrosis (VNN) disease resistance (Liu et al., 2016a, b) and four QTLs (PVE from 7.5 to 15.6%) associated with iridovirus were detected (Wang et al., 2017).

*Tenacibaculum maritimum* is a bacterial pathogen that poses a major threat to seabass farms in the South-East Asian region (Avendano-Herrera et al., 2006). In a collaborative study, we analyzed the microbiota of several tissues of commercially reared Asian seabass that showed symptoms of tenacibaculosis. We compared the microbial communities of diseased individuals to those of experimentally infected ones, using their healthy counterparts as controls in order to visualize the impact of tenacibaculosis on the fish microbiota. One of the bacterial genomes were sequenced, found to be different from isolates characterized so far and thus proposed as the type strain of *Tenacibaculum singaporense* sp. nov. (Miyake et al., 2020).

### Potential Improvements for the Asian Seabass Selection Program

A lot of important observations have been made by others and ourselves over those decades when the Asian seabass has been utilized for commercial aquaculture. In this chapter, we will provide a few suggestions for potential future improvements based on these observations.

**FIGURE 4:**
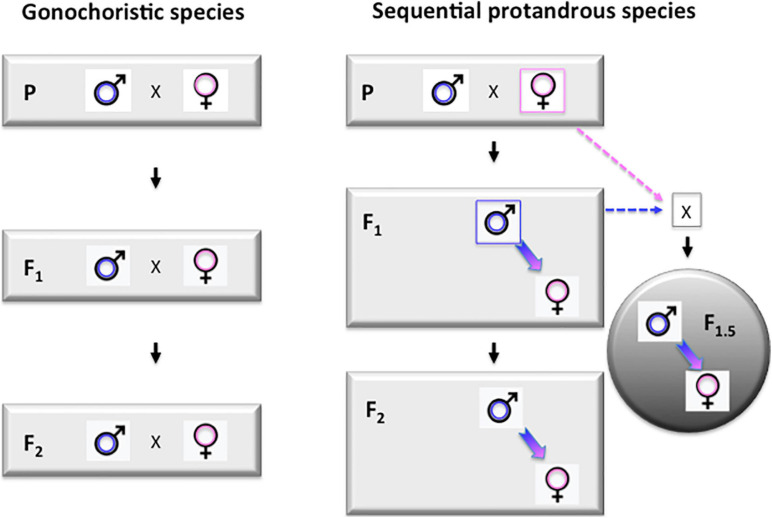
Protandrous sequential hermaphroditism opens up the possibility for producing “intermediate generations” during breeding programs. Left panel: schematic representation of the first three (P, F1, and F2) generations in a typical breeding program performed on a gonochoristic species, when the two sexes mature during the same period. Right panel: breeding scheme for a protandrous sequential hermaphrodite, like Asian seabass. All individuals first mature as males, making the production of so-called “intermediate generations” (labeled with gray circle on the right side) possible by crossing these young males with females from the previous generation. For an F1 × P cross the resulting generation is called F1.5 as they are F2s from the paternal side and F1s from the maternal one. Labels: P – parental (F0) generation; F1 and F2 – the first two generations of selected individuals. F1.5 – an “intermedate generation” produced by back-crossing protandrous hermaphroditic F1 males to founder (P) females.

Due the long generation time (3–4 years) of Asian seabass, the selection process is rather slow. However, the sequential hermaphroditic nature of the species (Moore, 1979; Davis, 1982; Guiguen et al., 1994) offers an interesting possibility to speed up the process. The essence of this approach is based on the formation of so-called “intermediate generations,” as proposed earlier by Robinson et al. (2010). Once the biggest individuals from the offspring groups of founding brooders reach the suitable size (around 50 cm standard length for individuals grown in seawater at tropical conditions) they start maturing as males. Once a few of these young males reach maturity, they can be back-crossed with the founding females to produce a new generation. We propose to call this F1.5 generation, as they are F2s on the paternal side, but only F1s on the maternal side (Figure 4). The performance of such F1.5 individuals is expected to be lower than the future F2s to be produced by the same F1 males crossed by future mature F1 females, but higher than the F1s. We have successfully performed several such crosses and grown the resulting offspring to market size. Such intermediate generations (i.e., F2.5, F3.5, etc.) can be produced after the appearance of each generation of males. In case of the seabass culture at tropical regions, where males start maturing between 1–1.5 years and natural sex reversal happens at 2.5–3 years, this trick allows farmers to tap into the benefits of the next generation 1.5 years earlier. Based on our current knowledge of the biology of the species (see, e.g., Davis, 1982), it seems likely that such “intermediate generations” are regularly produced in natural populations, when young males that matured in fresh water are swimming downstream to brackish waters to breed with females of earlier generations.

The fact that maleness precedes femaleness in Asian seabass opens up the way for expedited generation of near-isogenic lines without going through the laborious process of multi-generation inbreeding. Sperm samples can be cryopreserved from Asian seabass males and stored in liquid nitrogen for years (Leung, 1987). Once these individuals undergo the natural sex reversal and become mature females, their eggs can be stripped and fertilized with the frozen sperm collected earlier from the same individual as it was done in Eastern oyster (Yang et al., 2015) and shortnose sturgeon (Henne et al., 2006). Such “near-isogenic” lines produced from genetically distinct parents can later be used to create intraspecific hybrid lines that would likely yield well-performing offspring groups suitable for commercial purposes.

Setting up breeding groups for the mass crosses required patience and attention. Usually, after a few days the largest female brooder – or “alpha female” started to control the rest of the group and she started to determine the pecking order. When another female grew to a size similar to that of the alpha female, the leader began to challenge her. Aggressive behavior often resulted in chasing; tearing into the side of the body and gouging out scales, until the order is assumed or one of the females is removed. Transferring the “challenger” into an isolation tank often mitigated such situations. Brooders injured in the process were treated in “hospital tanks” where appropriate medication e.g., acriflavine, antibiotics were administered. Injured brooders usually recovered after a few weeks, but there were a few cases when the injuries suffered during the incident led to the loss of an individual. Based on the above, changing the composition of the breeding group usually required at least 2–3 weeks before they would settle and could be used for spawning.

During the selection process, we have occasionally formed breeding groups that contained a few siblings and half-sibs. Among the offspring of some these groups we have observed unwanted phenotypic signs – likely due to inbreeding effects – including partial or full loss of operculi on one or both sides and loss of dorsal fin. These could be typically eliminated by genotyping the affected individuals, matching them to their parents and removing one or both of the carriers from the spawning group.

We have shown earlier that Asian seabasses are not uniform across the whole geographic area occupied by them. The three different types (Indian, South-East Asian and Australian) show substantial differences at the level of genome and phenotype as well (Ward et al., 2005; Pethiyagoda and Gill, 2012; Vij et al., 2014, 2016). While natural populations might meet and potentially interbreed at bordering geographic areas, frequent transports of fertilized eggs or larvae between distant locations and even continents create additional opportunities for hybridizations within the species, but across the varieties (Alain Michel, personal communication). As the existing genomic platforms would make detailed analyses of potential connections between commercial stocks and natural populations around commercial facilities possible [for reviews on the topic see, e.g., Lorenzen et al. (2012) and Barrett et al. (2019)], we propose that such studies should be initiated to collect information about the frequency and extent of such hybridization events and their potential consequences.

## A Few Considerations for Food Fish Selection Programs

Although the first fish species were domesticated thousands of years ago and have been selected since then based on their phenotypes (for review see Balon, 2004), advanced selection programs supported by genetic/genomic platforms of food fishes have started much later than those of terrestrial farm animals. This delay offers certain opportunities, as researchers and farmers working on fish can utilize knowledge and approaches developed for those more advanced systems. The so-called “advantage of the follower” will allow aquatic selection programs for accelerated generation and rapid introduction of genomic platforms by following the examples of poultry, pigs and other farm animals (for a comparison of different selection programs applicable to aquaculture see Table 2).

**TABLE 2:** Comparison of different selection methods used in aquaculture.

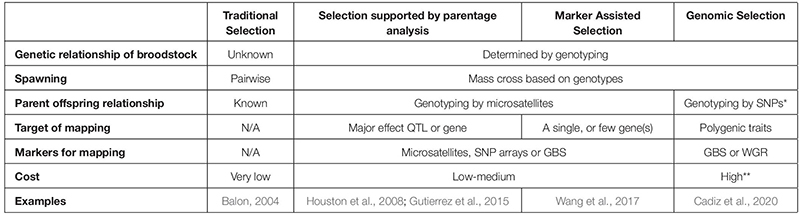

*GBS, genotyping-by-sequencing; N/A, not applicable; QTL, quantitative trait locus; SNP, single nucleotide polymorphism; WGR, whole genome re-sequencing. **The costs could be drastically reduced by genomic prediction using low density marker panels.

According to the survey of Chavanne et al. (2016), over three dozen of selective breeding programs are performed for the six most important food fish species in Europe, including the Atlantic salmon, rainbow trout, European seabass and common carp. There are several other selection programs worldwide, mostly in China, India, South-East Asia, Australia, New Zealand, and the United States. The estimated number of species cultured in these regions is substantially higher than in Europe. Examples include the GIFT project on Nile tilapia that supplied South-East Asia with elite offspring with improved growth rate and survival (Ponzoni et al., 2011), a 15-year selection program in striped catfish resulting in a 13.4% increase in growth rate (Vu et al., 2019), GS using extreme phenotypes for increased weight in large yellow croaker (Dong et al., 2016), and GS to increase the resistance of Japanese flounder against a bacterial pathogen (Liu et al., 2018). For comprehensive reviews on aquatic selection programs see Lind et al. (2012), Gjedrem and Rye (2018), and Houston et al. (2020).

What are the main advantages and disadvantages of selection programs in fishes? Fishes are extremely prolific species with thousands or even millions of offsprings per family. In addition, the generation time of most food fish species tends to be shorter, than those of large-bodied mammals, the initial goals of selection can be achieved earlier. The high number of offsprings allows for very high selection intensity [up to 2.51 in Nile tilapia (Bentsen et al., 2017) and 1.75 in channel catfish (Bosworth et al., 2020), possibly higher than that of poultry. As the initial costs of keeping and growing fish larvae and juveniles are typically lower than those of the poultry, most tools and selection procedures that are applicable during the early developmental phases can be used to test much larger offspring populations in fishes. On the other hand, the price of an offspring individual for most cultured fish species is typically very low, comparable to that of poultry. This prevents large-scale use of expensive genomic technologies (e.g., whole genome re-sequencing) and forces researchers to consider more cost-effective alternatives. Another important factor to consider is the early growth rate, as juveniles need to reach a certain size before they can be tagged physically or with RFID tags for subsequent re-identification following genotyping.

Aquatic selection programs can greatly benefit from the utilization of genetic/genomic platforms at multiple levels. The applicability of these tools depends on two factors: (i) the nature of the targeted trait; and (ii) the relative cost of the methods considered. When the trait is under the control of either a few major-effect loci or a few major QTL, they can be efficiently identified by more affordable MAS-based approaches [see e.g., Houston et al. (2008) and Moen et al. (2009)]. However, for polygenic traits a larger populations must be analyzed with a genome-wide coverage of markers, typically SNPs typed by a microarray or extracted from sequence data produced by GBS (Elshire et al., 2011). The initial costs for setting up some of these tools can be substantial and must be factored into the cost-benefit analysis of aquaculture breeding programs (Janssen et al., 2018). Whereas the most important terrestrial livestock species have several commercial SNP chips available, often with different densities of markers, aquaculture species – with the exception of salmon - have far more modest resources (for review see Houston et al., 2020). Due to these reasons, initially GS based on routinely performed high-density SNP typing was the privilege of those who worked on salmonids. A possible way to extend the benefits of these approaches to additional species with more modest resources is the use of the so-called genotype imputation. In this approach, tested first in salmon, only a few selected individuals (e.g., individuals from the breeding nucleus) are genotyped with high-density SNP arrays, whereas the rest are only tested at a much lower marker density (Tsai et al., 2017). Later, the use of low-density genotypes and the imputation were optimized (Tsairidou et al., 2020). Finally, the utility of low-to-medium density SNP panels (with the number of markers ranging 100 to 9,000) was tested in four datasets, analyzing five different traits (days to death, log length, weight, amoebic load, and gill score) in four species [salmon, common carp, gilthead seabream and Pacific oyster (Kriaridou et al., 2020)]. According to the results, typing with 1,000 SNPs allowed for a consistent pattern of genomic prediction accuracy in all cases. A very useful “byproduct” of these large-scale SNP-based typing experiments are high resolution genetic linkage maps created by the RAD-mapping technology (Hand et al., 2011; Campbell et al., 2014; Manousaki et al., 2015). Besides being useful for the dissection of regions containing genetic motifs of interests for selection, these maps are useful for the validations of assembled genomes as well (Lewin et al., 2009).

The ultimate genomic tool is a sequenced and assembled genome. Although a reference genome is not essential requirement for GS, the availability of information at such a high-resolution might exert obvious beneficial effects on such projects. The list of fish species with sequenced and assembled genome is rapidly expanding despite of the fact that they are more difficult to deal with due to their increased complexity stemming from a teleost-specific genome duplication (TGD) that happened in the common ancestor of today’s teleosts (Taylor et al., 2003; Christoffels et al., 2004). Currently the number of assembled teleost genomes stands around a hundred and it will likely double before the end of next year. Moreover, there are several large-scale efforts that target the genome sequencing and assembly of additional hundreds (Liu et al., 2017) or even tens of thousands of vertebrates, among them many fishes [see e.g. Vertebrate Genomes Project (VGP), 2021]. Selection programs on a tighter budget can focus on the transcriptome instead of the genome, as the former can be deciphered from a reasonable cost and it provides information on the protein-coding regions. There are an estimated 25,000–28,000 protein-coding genes in a typical, diploid teleost genome, about 30% more than in a typical terrestrial mammal (Wittbrodt et al., 1998; Steinke et al., 2006). Further improvements of the transcriptome are possible through approaches allowing for the inventory of all transcripts produced from a locus (see, e.g., Zhao et al., 2019) and the development of the capability of exome sequencing [see, e.g., Teer and Mullikin (2010), Bamshad et al. (2011) and Bao et al. (2014)]. They can provide more detailed information about the regions that house the protein-coding genes and important non-coding sequences associated with them as well. This could prove to be very useful for the identification of candidate genes.

## Conclusion

Aquatic selection programs have entered the era, when their dependence on genomic tools and platforms will drastically increase. By following the footsteps of those working with terrestrial farm animals, researchers have been developing genomic toolboxes of an increasing number of food fish species used in aquaculture production.

This review describes the history of the selection program performed on Asian seabass in Singapore over the last 15 years. It covers the protocols applied at the farm, the full genomic toolbox developed for the species and their application in different studies. As this might be the first selection program performed on a tropical, sex changing predator, some of the unique aspects stemming from these features are also described. A few considerations for aquatic selection programs are also provided in the hope that they will be useful for those who are in the planning phase of such projects.

We hope that our review will be useful not only for researchers and farmers working with Asian seabass, but potentially for those who face similar decisions in the work with other, lesser known tropical food fishes as well.

## Author Contributions

LO and LV conceived the idea of writing this review. LV wrote the parts on terrestrial farm animals and contributed to the part on potential solutions for problems. XS wrote the part on the farm-based disease challenges, whereas NP contributed the part on practical observations from the farm. LO wrote the remaining parts. LV and LO synchronized all sections and finalized the MS. All authors critically revised the manuscript for intellectual content.

## Conflict of Interest

The authors declare that the research was conducted in the absence of any commercial or financial relationships that could be construed as a potential conflict of interest.

## Supplementary Material

Supplementary Table 1 The list of aquatic invertebrate and fish species mentioned in the review.
