# Supplementary material for: Toward Genome-Based Selection in Asian Seabass: What Can We Learn From Other Food Fishes and Farm Animals?
Source: Front Genet. 2021 Apr 21;12:506754. doi: 10.3389/fgene.2021.506754 (PMC8097054; doi:10.3389/fgene.2021.506754)
Supplement: Supplementary Table 1 — The list of aquatic invertebrate and fish species mentioned in the review. [file Table_1.pdf]

Supplementary Table 1: The list of aquatic invertebrate and fish species mentioned in the review

Common name	Latin name
Asian seabass	*Lates calcarifer*, Bloch 1790
Atlantic cod	*Gadus morhua*, L. 1758
Atlantic salmon	*Salmo salar,* L. 1758
Channel catfish	*Ictalurus punctatus,* Rafinesque 1818
Columbia River redband trout*	*Oncorhynchus mykiss gairdneri*, Richardson 1836
Common carp	*Cyprinus carpio*, L. 1758
Eastern oyster	*Crassostrea virginica*, Gmelin 1791
European seabass	*Dicentrarchus labrax*, L. 1758
Gilthead sea bream	*Sparus aurata*, L. 1758
Japanese flounder	*Paralichthys olivaceus,* Temminck & Schlegel 1846
Large yellow croaker	*Larimichthys crocea,* Jordan & Seale 1905
Nile tilapia	*Oreochromis niloticus,* Günther 1889
Pacific oyster	*Magallana gigas*, Thunberg 1793
Rainbow trout	*Oncorhynchus mykiss,* Walbaum 1792
Shortnose sturgeon	*Acipenser brevirostrum*, Lesueur 1818
Striped catfish	*Pangasianodon hypophthalmus,* Sauvage 1878

* A subspecies of rainbow trout.
